# Early diagnosis of miliary tuberculosis in a hemodialysis patient by combining two interferon-γ-release assays: a case report

**DOI:** 10.1186/s12882-020-01875-w

**Published:** 2020-06-04

**Authors:** Florence Bonkain, Dieter De Clerck, Violette Dirix, Mahavir Singh, Camille Locht, Françoise Mascart, Véronique Corbière

**Affiliations:** 1 Department of Nephrology, Universitair Ziekenhuis Brussel (UZ Brussel), Brussels, Belgium; 2 Laboratory of Vaccinology and Mucosal Immunity, Université Libre de Bruxelles (U.L.B.), Brussels, Belgium; 3 Lionex Diagnostics and Therapeutics, Braunschweig, Germany; 4 U1019 – UMR 8204 – CIIL - Center for Infection and Immunity of Lille, Univ. Lille, CNRS, Inserm, CHU Lille, Institut Pasteur de Lille, F-59000 Lille, France

**Keywords:** *Mycobacterium tuberculosis* infection, Diagnosis, Interferon-gamma release assay, Hemodialysis

## Abstract

**Background:**

Patients with end-stage renal disease undergoing chronic hemodialysis (HD) are at high risk to develop tuberculosis (TB) associated with a high mortality rate. TB diagnosis is often delayed due to non-specific symptoms, frequent extra-pulmonary manifestations, and rare microbiological confirmation. This case report illustrates the clear added value of combined interferon-γ -release assays (IGRA) in response to different mycobacterial antigens for an early diagnosis of TB in HD patients.

**Case presentation:**

We report the case of an Egyptian patient under chronic HD treatment, who presented with recurrent episodes of fever and myalgia of unknown origin, associated with an important inflammatory syndrome. These episodes resolved partially or completely within less than 1 month without any treatment but recurred 10 times within 3 years. Chest Computed Tomography and 18F-fluorodeoxyglucose Positron Emission Tomography/Computed Tomography (18FDG PET-CT) demonstrated several active mediastinal lymphadenopathies. TB was the first suspected diagnosis but cultures and polymerase chain reaction (PCR) remained negative on a mediastinal lymph node aspiration. In contrast, the results from two different IGRA performed on blood were highly suggestive of TB disease. Several granulomas, some of them with central non-caseating necrosis, were demonstrated on a pulmonary nodule obtained by thoracoscopic resection, but PCR and culture remained negative for *M. tuberculosis.* Three years after the initial symptoms, a new PET-CT revealed a retro-clavicular lymphadenopathy in addition to the mediastinal lymphadenopathies, and the *M. tuberculosis* culture performed on the resected lymphadenopathy was positive. Antibiotic treatment for TB was started and resulted in a clear improvement of the patient’s clinical condition, allowing him to successfully receive a renal graft.

**Conclusions:**

In view of the high frequency of TB in patients undergoing chronic HD and of the limitations of the classical diagnosis procedures, nephrologists have to diagnose TB mostly on clinical suspicion. We demonstrate here that the use of a combined IGRA to two different mycobacterial antigens may significantly raise the index of suspicion and help clinicians to decide starting anti-TB treatment in HD patients.

## Background

*Mycobacterium tuberculosis* infection remains a worrying public health problem among end-stage renal disease (ESRD) patients due to the high incidence of tuberculosis (TB) [1] and its association with significant mortality in these patients [2–4]. The incidence of active TB (aTB) is 6- to 25-fold higher in hemodialysis (HD) patients than in the general population, with 2- to 3-fold higher mortality rates during treatment [5–7]. Moreover, the diagnosis is often delayed or missed due to atypical clinical presentation of aTB in ERSD, as cardinal symptoms such as cough, night sweats and weight loss are often mild or even completely absent [7]. Extra-pulmonary manifestations characterized by non-specific symptoms occur in more than half of the patients [8, 9]. The most frequent are TB lymphadenitis (TBLA) [10], followed by peritoneal, pericardial, bone and urinary infections.

Due to particularly high mortality rates, early diagnosis of aTB is extremely important for these patients. However, direct evidence of pulmonary aTB, as provided by a positive *M. tuberculosis* culture, is rarely obtained in patients under HD, and is even rarer in HD patients with extra-pulmonary aTB [11, 12]. In addition, complementary, more invasive investigations, such as biopsies, to detect *M. tuberculosis* are often not carried out in ESRD, due to the increased risk in these patients of bleeding complications because of platelet dysfunction, disturbed coagulation and heparin administration during HD.

Most aTB cases in HD patients result from reactivation of a latent TB infection (LTBI) rather than from a new infection, and direct transmission of aTB between HD patients is rare [5]. It is therefore highly recommended to detect LTBI in these patients in order to offer prophylactic antibiotherapy [13–15]. The tuberculin skin test (TST) remains the gold standard for LTBI detection in many countries, especially in those with low BCG vaccination coverage. However, the TST is not optimal in ESRD patients under HD, as it is associated with high rates of false-negative results in patients with impaired cell-mediated immunity due to uremia and/or to immunosuppressive medications [16, 17]. Detection of LTBI is these patients remains feasible thanks to in-vitro tests measuring the interferon-gamma (IFN-γ) release by blood cells incubated with *M. tuberculosis* antigens, referred to as IFN-γ release assays (IGRA). Two such assays are marketed, the QuantiFERON-TB Gold In-Tube (QFT) (Qiagen, US) and the T-SPOT TB (Oxford Immunotec, UK). Both were reported to be more sensitive than TST for the diagnosis of infected HD patients [18–20] and to be more specific than TST, particularly in BCG-vaccinated patients [21]. The major drawback of these tests is the fact that they do not provide discrimination between aTB and LTBI subjects, a problem of major importance in patients with insidious and atypical presentation of aTB. Alternatively, combining the analysis of the IFN-γ responses to two different mycobacterial antigens, the early-secreted antigen target-6 (ESAT-6), and the heparin-binding hemagglutinin (HBHA) may help to differentiate LTBI from aTB [22, 23]. High ESAT-6-induced IFN-γ secretion with no or low HBHA-induced IFN-γ secretion is associated with aTB or with a risk to develop aTB, whereas an isolated IFN-γ response to HBHA is a hallmark of LTBI with a limited risk of reactivation of the infection. In addition, using IGRA based on HBHA-induced IFN-γ secretion is more sensitive than the QFT for the detection of LTBI in ESRD patients undergoing HD [24].

We report here the case of an Egyptian HD patient presenting with recurrent fever episodes associated with myalgia and general malaise. aTB was finally diagnosed by a positive *M. tuberculosis* culture on a cervical lymph node biopsy, while the results of a combined ESAT-6- and HBHA-IGRA already strongly suggested aTB 3 years before the final diagnosis.

## Methodology

This case report has been written according the CARE guidelines. Written consent of the patient was obtained before writing the manuscript and approval was obtained by the ethic committee “Commissie Medische Ethiek” from the “Vrije Universiteit Brussel” (CME 2019–395).

## Case presentation

A 57 year old man born in Egypt started chronic HD treatment in 2011 in Belgium for ESRD due to chronic pyelonephritis and kidney stones. The patient came to live in Belgium in 1997 but he often travelled to Egypt. The medical history included ischemic cardiomyopathy, type 2 diabetes mellitus, and urinary shistosomiasis. An arterio-venous graft was placed due to the irreversible occlusion of the fistulae, and the patient had undergone surgery for urethral stenosis. His home medication consisted of aspirin, bisoprolol, simvastatine, furosemide, oral antidiabetic medication and a phosphate-binder, whereas erythropoietin, iron substitution and vitamin B complex were administrated during HD sessions.

**Fig. 1:**
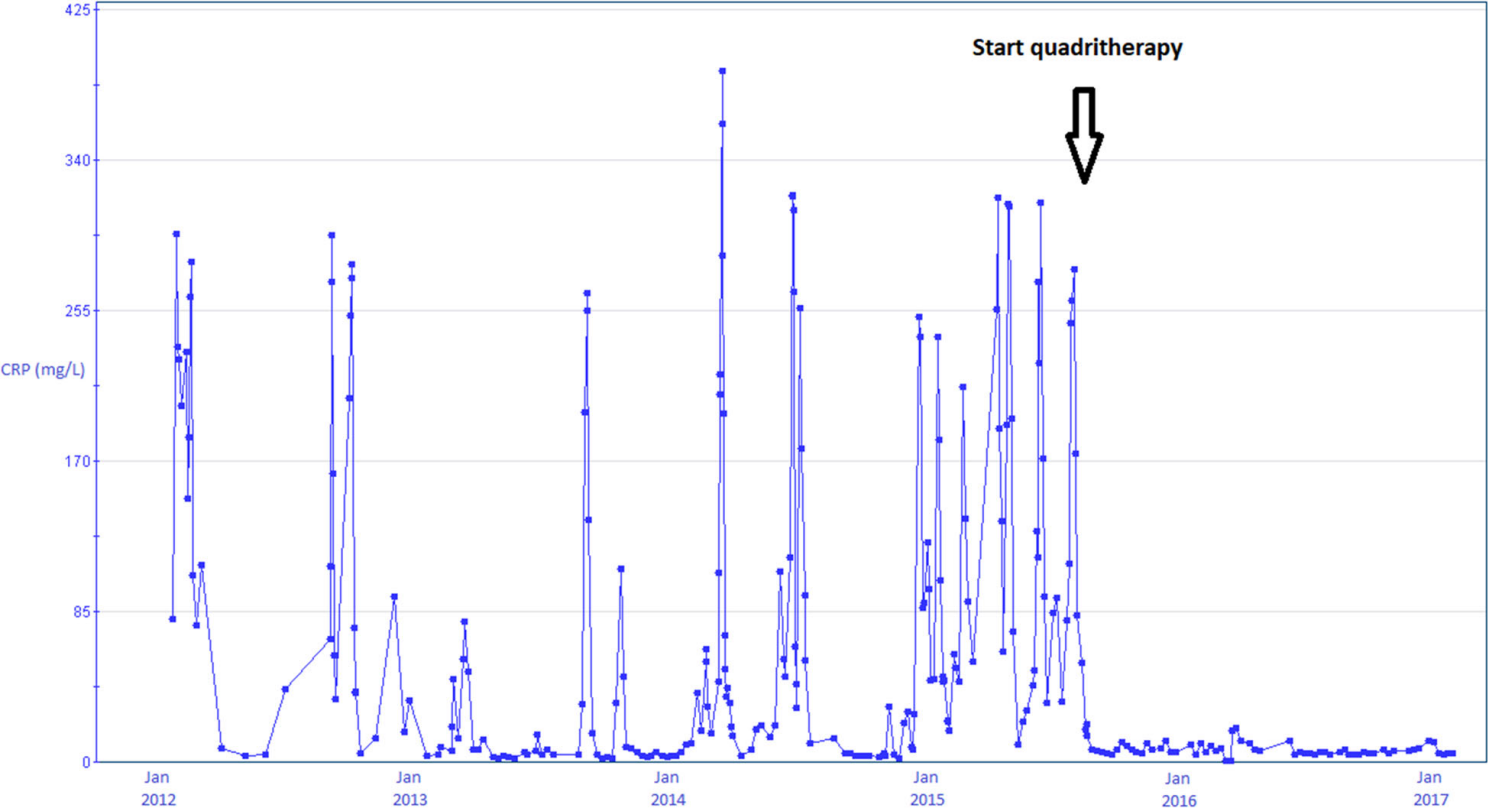
Evolution of CRP levels from January 2012 until January 2017. CRP concentrations were measured in blood at multiple time-points and are reported in mg/L. Each dot indicates a result of CRP concentration, and the different results are linked by a line. The vertical arrow indicates the time point of the start of the anti-TB quadritherapy

Between February 2012 and July 2015, the patient presented with several episodes of recurrent fever with myalgia after coming back from his journeys in Egypt. He had no diarrhea, nor any urinary symptoms or respiratory complaints, and reported no insect or dog bites. His physical examination was normal, but each episode of fever and myalgia was accompanied by high C-reactive protein (CRP) blood levels (Fig. 1), while liver enzyme concentrations remained normal. At each episode, both the inflammatory syndrome and the clinical complaints decreased partially or completely within less than 1 month, as illustrated in Fig. 1, without any explanation or specific treatment. The patient was hospitalized at each episode for exhaustive infectious evaluations, and blood cultures were sampled at each HD session, as well as during chills and/or peaks of temperature. All bacteriological blood cultures remained negative, as well as viral serologies for HIV, hepatitis A, B and C. Chest radiography did not show any infiltrate, cavity or calcification, whereas a Computed Tomography (CT) scan demonstrated a pulmonary infiltrate in the left lower lobe, and a mediastinal adenopathy. Ultrasounds of the arterio-venous graft excluded a collection near the prosthetic material. Given the impaired immunity related to the ESRD, the frequent travels to Egypt and the past history of schistosomiasis, a recurrence of parasitic infection was suspected and the patient received an empirical treatment based on praziquantel, despite the absence of parasitological proof of Shistosomiasis. A second CT scan performed 3 months later confirmed the presence of a pulmonary infiltrate in the left lower lobe and demonstrated several mediastinal lymphadenopathies. A multitude of differential diagnoses were evoked, including infectious diseases (*M. tuberculosis* or atypical mycobacteria, herpes virus, histoplasmosis, toxoplasmosis, leishmaniosis, trypanosomiasis, filariasis, rickettsiosis), systemic diseases (sarcoidosis, systemic lupus erythematosus, dermatomyositis, Sjogren, Sharp, Castelman syndrome, histiocytosis, anthraco-silicosis) and neoplastic diseases (Hodgkin disease or other lymphoma, leukemia, metastasis of a solid cancer). Viral, parasitic and auto-immune serologies were all negative as were several searches for parasites in the feces. An endobronchial ultrasound-guided trans-bronchial needle aspiration (EBUS) of a mediastinal lymph node was performed, but provided no element in favor of any infectious or neoplastic disease, although sinus histiocytosis was demonstrated. To exclude *M. tuberculosis* infection, PCR and cultures were performed on broncho-alveolar lavage fluid and on the mediastinal lymph node aspiration, and they were negative. A TST was not performed because of its poor negative predictive value in HD patients [17, 20]. Instead, we performed two well-standardized home-made IGRA on peripheral blood mononuclear cells (PBMC) and compared the IFN-γ secretion induced by ESAT-6 with that induced by HBHA. The secretion of IFN-γ upon stimulation with purified protein derivative (PPD or tuberculin) was used as a positive control of mycobacterial infection [22–24]. In addition, staphylococcal enterotoxin B (SEB) was used as a positive control for T cell reactivity, while the negative control contained no added stimulant. These IGRA performed in October 2012 resulted in a very high level of IFN-γ secretion in response to ESAT-6 and a low level in response to HBHA (Table 1). Combined with the clinical presentation, these results were highly suggestive of aTB, but this diagnosis was at that time not retained because of the negative microbiological results. In October 2012, after the same recurrent complaints, a 18F-fluorodeoxyglucose Positron Emission Tomography/Computed Tomography (18FDG PET-CT) was performed with the hope to identify a lesion accessible for biopsy. It only confirmed the existence of several FDG-active mediastinal lymphadenopathies accompanied by a small subpleural infiltrate in the left lower lobe. Several lymph nodes were removed by mediastinoscopy but Ziehl-Neelsen acid-fast stain was negative as were *M. tuberculosis* cultures and PCR.

**Table 1:** evolution of the symptoms, the treatment and IGRA results

Dates	October 2012	April 2013	July 2015	January 2016	February 2018	May 2018
Symptoms	Fever, myalgia	Fever, myalgia	Fever, myalgia	Absent	Absent	absent
PET-CT	Mediastinal adenopathies	Mediastinal adenopathies+ pulmonary nodules	Cervical, retro-clavicular, mediastinal, retroperitoneal, hepatic hilar adenopathies + pulmonary nodule	NA	NA	NA
*M. tuberculosis* culture/PCR	Negative	Negative	Positive	NA	NA	NA
Treatment						
Anti-TB	No	No	Yes	Yes	No	No
Kidney graft	No	No	No	No	Yes	Yes
Immuno-suppression	No	No	No	No	Yes	Yes
IGRA results (pg/ml)						
Neg control	0	NA	NA	0	NA	0
Pos control	27,464			34,803		4767
PPD	28,164			23,611		202
HBHA	2862			6362		0
ESAT-6	13,166			6118		85

*PET-CT:* Positron Emission Tomography/Computed Tomography; *Neg*.: negative; *Pos*.: positive; *NA*: not available; *IGRA*: interferon--release assay; *SEB*: staphylococcal enterotoxin B; *PPD*: purified protein derivative; *HBHA*: heparin-binding-hemagglutinin; *ESAT-6*: early-secreted-antigen target-6; *IGRA* positivity limits: Pos control > 2000 pg/ml - PPD > 200 pg/ml - HBHA > 50 pg/ml – ESAT-6 > 50 pg/ml

In April 2013, fever and myalgia reappeared and a second PET-CT was performed, which demonstrated de novo FDG-positive pulmonary nodules, in addition to the preexisting and stable mediastinal lymphadenopathies. Thoracoscopic resection of an apical pulmonary nodule was performed, which showed several granulomas, a few of which with central non-caseating necrosis. However, due to persistent negativity of the *M. tuberculosis* cultures and PCR, the clinicians did not prescribe anti-TB treatment.

**Fig. 2:**
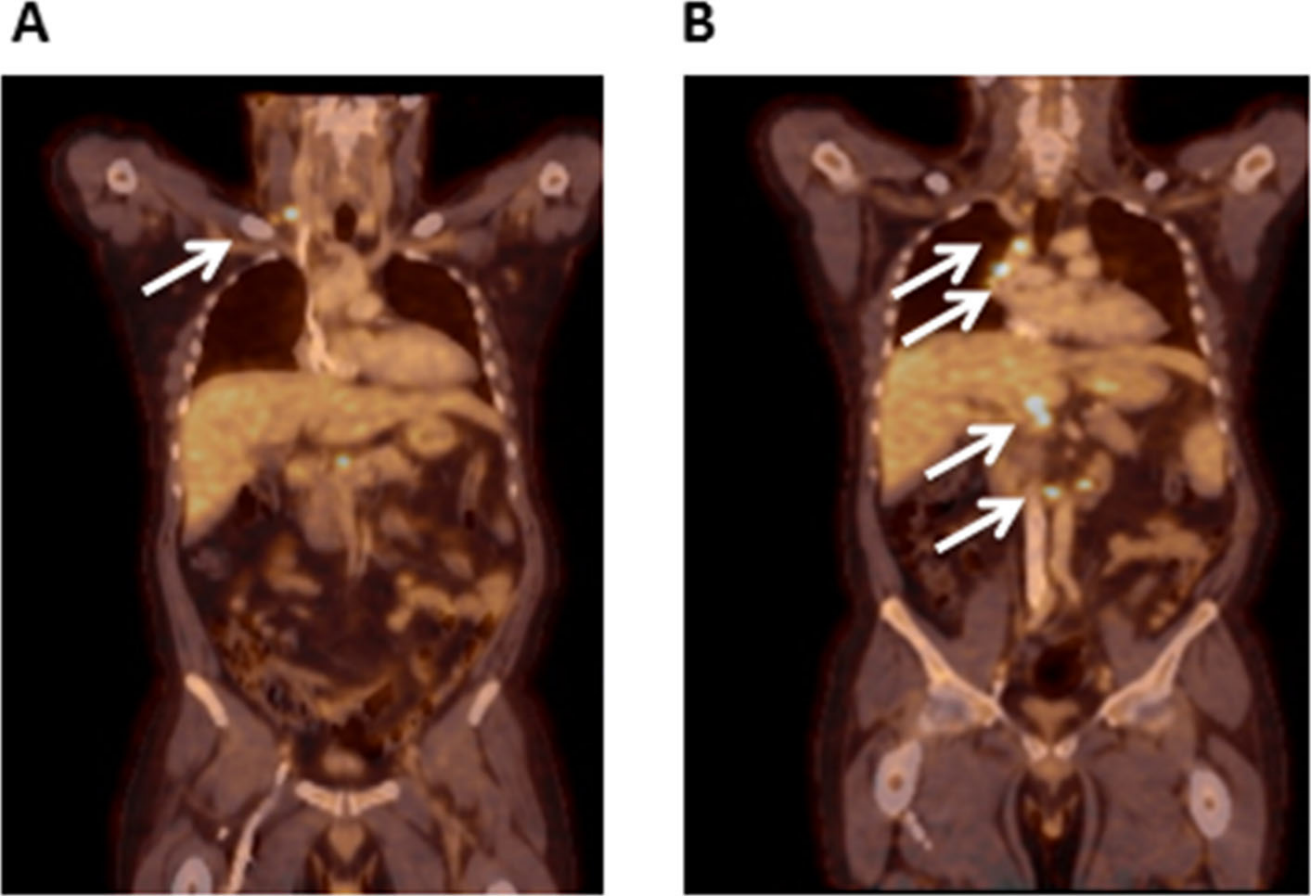
PET-CT analysis in July 2015. Cervical lymphadenopathy (**a**) and mediastinal, retroperitoneal and liver hilar lymph nodes (**b**) are indicated by the arrows

In July 2015, a new PET-CT was performed following a recurrent fever episode, and it highlighted now cervical, mediastinal, retroperitoneal and hepatic hilar FDG-active lymph nodes (Fig. 2). The patient underwent a resection of a retro-clavicular lymphadenopathy for further search of *M. tuberculosis*, and the Ziehl-Neelsen acid-fast stain was positive. After 2 weeks, the culture was positive for *M. tuberculosis*, confirming the diagnosis of TBLA, and quadritherapy was started. Adherence and tolerability to the treatment were assessed twice weekly during the HD session. Since the patient did not have a family doctor, all the prescriptions were given at regular basis by the nephrologists. The treatment shifted after 2 weeks to a tritherapy (tebrazid, rifadine and myambutol) for 14 months, as isoniazid had to be stopped because of encephalopathy. The inflammatory syndrome disappeared completely under treatment (Fig. 1), as well as the patient’s symptoms. The IGRA performed after 6 months of treatment indicated an increased IFN-γ response to HBHA and a decreased response to ESAT-6 compared to the first IGRA, suggesting a favorable response to treatment [25]. The patient gained 8 kg during his treatment and could successfully undergo kidney transplantation in February 2018. Three months after the transplantation, the IGRA results were doubtful in response to PPD, negative in response to HBHA, but still slightly positive in response to ESAT-6 (Table 1). The patient was at that time under immunosuppressive treatment. However, since he nevertheless secreted measurable levels of IFN-γ secretion in response to SEB, we considered him as cured from the *M. tuberculosis* infection.

## Discussion and conclusions

This case report illustrates the potential of a combined IGRA to two different mycobacterial antigens to predict the risk of LTBI progression and to diagnose aTB in HD patients. The very high IFN-γ secretion induced by ESAT-6 compared with a lower IFN-γ response to HBHA identified this patient as LTBI with a high risk to develop aTB or as a patient with aTB [22, 23]. High IFN-γ responses to ESAT-6 are more often associated with aTB than with LTBI, whereas the reverse is true for IFN-γ responses to HBHA [23, 26]. *M. tuberculosis*-infected subjects may therefore be classified in 3 groups according to their IFN-γ responses to these two antigens: positive to only HBHA, to only ESAT-6 or to both antigens. Elevated IFN-γ responses to ESAT-6 with lower or undetectable IFN-γ responses to HBHA suggest aTB [22, 27].

The initial IGRA results obtained for this patient coming from a high TB incidence country and merely complaining of episodes of fever with myalgia accompanied by an inflammatory syndrome suggested poorly controlled *M. tuberculosis* infection. The demonstration of several FDG-active mediastinal lymphadenopathies by 18FDG PET-CT led to a possible diagnosis of aTB lymphadenitis. However, as the EBUS had not provided direct proof of TB by positive PCR or culture, TB diagnosis was initially rejected. The resection of a mediastinal lymph node and of a pulmonary nodule also did not provide direct proof of a mycobacterial infection. The evidence was provided only 2 years later after the resection of a retro-clavicular adenopathy. Even though we have no formal proof that the patient already suffered from aTB at the beginning of the symptoms 3 years earlier, the recurrent episodes of fever with severe myalgia associated with very high CRP concentrations that resolved only during the course of the TB treatment, strongly suggest that the patient initially already presented with TBLA, and that the diagnosis was missed because formal microbiological proof of aTB was lacking.

TBLA is the most common manifestation of extra-pulmonary TB with a 15–20% prevalence rate in TB endemic countries [28, 29]. Whereas a similar frequency of TBLA was reported among HD patients in India [30], TBLA represented 26.3% of TB cases among HD patients in Sudan [8]. However, in adults, TBLA most often affects cervical lymph nodes, whereas hilar and mediastinal TBLA is a common feature of pediatric cases and is rather a characteristic of primary TB than of post-primary TB [31]. Both in children and in adults, TB diagnosis of patients with mediastinal lymphadenopathies remains a challenge, as not only the clinical signs and symptoms but also the computed tomography characteristics are not specific for aTB. Even though the EBUS provides access to mediastinal adenopathies to perform microbiological cultures and / or PCR, the diagnostic performance of this approach for aTB lies only between 70 and 90% [32–34]. A formal diagnosis of TBLA based on a positive *M. tuberculosis* culture or PCR is therefore often delayed, increasing the likelihood of serious complications and death.

This case report therefore suggests that nephrologists should reconsider the criteria they use to diagnose TBLA in their HD patients. Early diagnosis, especially in young patients waiting for a kidney graft, may drastically reduce the morbidity and the mortality associated with TB and may allow HD patients suspected to suffer from aTB to be grafted earlier. A high degree of suspicion should arise for ERSD patients presenting with general symptoms such as fever, weight loss, and/or lymphadenopathy [11]. We demonstrate here that even when formal microbiological proof of aTB is lacking in most cases, abnormal results of the two combined IGRA described here should further alert the clinician, as they are highly suggestive of aTB. This new criterium should therefore be considered to enable early treatment of HD patients waiting for a renal graft, which became possible in the patient reported here only after successful treatment of the TBLA.

## Data Availability

The datasets analysed during the current study are available from the corresponding author on reasonable request.

## Abbreviations

aTB: Active tuberculosis
BCG: Bacillus Calmette et Guérin
CRP: C-reactive protein
EBUS: endobronchial ultrasound-guided transbronchial needle aspiration
ESAT-6: Early-secreted antigen target-6
ESRD: end stage renal disease
HBHA: Heparin-binding hemagglutinin
HD: Hemodialysis
IFN- γ: Interferon-gamma
IGRA: IFN-γ release assay
LTBI: latent tuberculosis infection
*M. tuberculosis*: *Mycobacterium tuberculosis*
PBMC: peripheral blood mononuclear cells
PCR: Polymerase chain reaction
PET-CT: Positron Emission Tomography/Computed Tomography
PPD: Purified protein derivative
QFT: QuantiFERON-TB Gold In-Tube
SEB: Staphylococcal enterotoxin B
TB: Tuberculosis
TBLA: Tuberculous lymphadenitis
TST: Tuberculin skin test

## References

[1] Al-Efraij K, Mota L, Lunny C, Schachter M, Cook V, Johnston J (2015). Risk of active tuberculosis in chronic kidney disease : a systematic review and meta-analysis. Int J Tuberc Lung Dis.

[2] Wakasugi M, Kawamura K, Yamamoto S, Kazama JJ, Narita I (2012). High mortality rate of infectious diseases in dialysis patients : a comparison with the general population in Japan. Ther Apher Dial.

[3] Klote MM, Agodoa LY, Abbott KC (2006). Risk factors for mycobacterium tuberculosis in US chronic dialysis patients. Nephrol Dial Transplant.

[4] Bai KJ, Huang KC, Lee CH, Tang CH, Yu MC, Sue YM (2017). Effect of pulmonary tuberculosis on clinical outcomes of long-term dialysis patients : pre- and post-DOTS implementation in Taiwan. Respirology..

[5] Okada RC, Barry PM, Skarbinski J, Chitnis AS (2018). Epidemiology, detection, and management of tuberculosis among end-stage renal disease patients. Infect Control Hosp Epidemiol.

[6] Malik GH, Al-Mohaya SA, Al-Harbi AS (2003). Spectrum of tuberculosis in dialysis patients in Saudi Arabia. Saudi J Kidney Transpl.

[7] Malik GH, Al-Harbi AS, Al-Mohaya S (2002). Eleven years of experience with dialysis associated tuberculosis. Clin Nephrol.

[8] Banaga AS, Siddiq NK, Alsayed RT, Babiker R, Elmusharaf K (2016). Prevalence and presentation of tuberculosis among hemodialysis patients in Khartoum. Sudan Saudi J Kidney Transpl.

[9] Mitwalli A (1991). Tuberculosis in patients on maintenance dialysis. Am J Kidney Dis.

[10] Abdelrahman M, Sinha AK, Karkar A (2006). Tuberculosis in end-stage renal disease patients on hemodialysis. Hemodial Int.

[11] Segall L, Covic A (2010). Diagnosis of tuberculosis in dialysis patients : current strategy. Clin J Am Soc Nephrol.

[12] Chuang FR, Lee CH, Wang IK, Chen JB, Wu MS (2003). Extrapulmonary tuberculosis in chronic hemodialysis patients. Ren Fail.

[13] Ostermann M, Palchaudhuri P, Riding A, Begum P, Milburn HJ (2016). Incidence of tuberculosis is high in chronic kidney disease patients in south East England and drug resistance common. Ren Fail.

[14] Horsburgh CR (2004). Priorities for the treatment of latent tuberculosis infection in the United States. N Engl J Med.

[15] Li SY, Chen TJ, Chung KW (2011). Mycobacterium tuberculosis infection of end-stage renal disease patients in Taiwan: a nationwide longitudinal study. Clin Microbiol Infect.

[16] Centers for Disease Control (CDC). Screening for tuberculosis and tuberculosis infection in high-risk populations. Recommendations of the Advisory Council for the Elimination of Tuberculosis. MMWR Recomm Rep. 1995 Sep 8; 44 (RR-11):19–34. 7565540

[17] Soysal A, Toprak D, Koc M, Arikan H, Akoglu E, Bakir M (2012). Diagnosing latent tuberculosis infection in haemodialysis patients : T-cell based assay (T-SPOT.TB) or tuberculin skin test ?. Nephrol Dial Transplant.

[18] Lee SS, Chou KJ, Dou HY (2010). High prevalence of latent tuberculosis infection in dialysis patients using the interferon-gamma release assay and tuberculin skin test. Clin J Am Soc Nephrol.

[19] Sester M, Sester U, Clauer P (2004). Tuberculin skin testing underestimates a high prevalence of latent tuberculosis infection in hemodialysis patients. Kidney Int.

[20] Passalent L, Khan K, Richardson R, Wang J, Dedier H, Gardam M (2007). Detecting latent tuberculosis infection in hemodialysis patients: a head-to-head comparison of the T-SPOT.TB test, tuberculin skin test, and an expert physician panel. Clin J Am Soc Nephrol.

[21] Doan TN, Eisen DP, Rose MT, Slack A, Stearnes G, McBryde ES (2017). Interferon-gamma release assay for the diagnosis of latent tuberculosis infection: a latent-class analysis. PLoS One.

[22] Corbière V, Pottier G, Bonkain F (2012). Risk stratification of latent tuberculosis defined by combined interferon gamma release assays. PLoS One.

[23] Mascart F, Locht C (2015). Integrating knowledge of *Mycobacterium tuberculosis* pathogenesis for the design of better vaccines. Expert Rev Vaccines.

[24] Dessein R, Corbière V, Nortier J (2013). Heparin-binding haemagglutinin, a new tool for the detection of latent mycobacterium tuberculosis infection in hemodialysis patients. PLoS One.

[25] Masungi C, Temmerman S, Van Vooren JP (2002). Differential T and B-cell responses against *Mycobacterium tuberculosis* heparin-binding hemagglutinin in infected healthy individuals and tuberculosis patients. J Inf Dis.

[26] Hougardy JM, Schepers K, Place S (2007). Heparin-binding-hemagglutinin-induced IFN-gamma release as a diagnostic tool for latent tuberculosis. PLoS One.

[27] Delogu G, Chiacchio T, Vanini V (2011). Methylated HBHA in M. smegmatis discriminates between active and non-active tuberculosis disease among RD1-responders. PLoS One.

[28] Richardson RM (2012). The diagnosis of tuberculosis in dialysis patients. Semin Dial.

[29] Lee JY (2015). Diagnosis and treatment of extrapulmonary tuberculosis. Tuberc Respir Dis.

[30] Vikrant S (2019). Tuberculosis in dialysis : clinical spectrum and outcome from an endemic region. Hemodial Int.

[31] Nin CS, de Souza VV, do Amaral RH et al. Thoracic lymhadenopathy in benign disease : a state of the art review. Respir Med 2016;112:10–17. 10.1016/j.rmed.2016.01.021 26860219

[32] Navani N, Molyneaux PL, Breen RA (2011). Utility of endobronchial ultrasound-guided transbronchial needle aspiration in patients with tuberculous intrathoracic lymphadenopathy: a multicentre study. Thorax.

[33] Harris RM, Arnaout R, Koziel H, Folch E, Majid A, Kirby JE (2016). Utility of microbiological testing of thoracic lymph nodes sampled by endobronchial ultrasound-guided transbronchial needle aspiration (EBUS-TBNA) in patients with mediastinal lymphadenopathy. Diagn Microbiol Infect Dis.

[34] Lin CK, Keng LT, Lim CK (2019). Diagnosis of mediastinal tuberculous lymphadenitis using endobronchial ultrasound-guided transbronchial needle aspiration with rinse fluid polymerase chain reaction. J Formos Med Assoc.

